# Assessing the Effects of Software Platforms on Volumetric Segmentation of Glioblastoma

**DOI:** 10.17756/jnpn.2016-008

**Published:** 2016-07-20

**Authors:** William D. Dunn, Hugo J.W.L. Aerts, Lee A. Cooper, Chad A. Holder, Scott N. Hwang, Carle C. Jaffe, Daniel J. Brat, Rajan Jain, Adam E. Flanders, Pascal O. Zinn, Rivka R. Colen, David A. Gutman

**Affiliations:** 1 Departments of Biomedical Informatics and Neurology, Emory University School of Medicine, Atlanta, GA, USA; 2 Departments of Radiation Oncology and Radiology, Dana-Farber Cancer Institute, Brigham and Women’s Hospital, Harvard Medical School, Boston, MA, USA; 3 Department of Biostatistics & Computational Biology, Dana-Farber Cancer Institute, Boston, MA, USA; 4 Department Winship Cancer Institute, Emory University, Atlanta, GA, USA; 5 Department Biomedical Engineering, Georgia Institute of Technology/Emory University, Atlanta, GA, USA; 6 Department of Radiology and Imaging Sciences, Emory University School of Medicine, Atlanta, GA, USA; 7 Department of Diagnostic Imaging Department, St. Jude Children’s Research Hospital, Memphis, TN, USA; 8 Department of Radiology, Boston University School of Medicine, Boston, MA, USA; 9 Department of Pathology and Laboratory Medicine, Emory University School of Medicine, Atlanta, GA, USA; 10 Departments of Radiology and Neurosurgery, NYU School of Medicine, New York, NY, USA; 11 Department of Neuroradiology, Thomas Jefferson University Hospitals, Philadelphia, PA, USA; 12 Department of Neurosurgery, The University of Texas MD Anderson Cancer Center, Houston, TX, USA; 13 Department of Diagnostic Radiology, The University of Texas MD Anderson Cancer Center, Houston, TX, USA

**Keywords:** Imaging-genomics, GBM, MRI, Cancer, Computational science, 3D Slicer

## Abstract

**Background:**

Radiological assessments of biologically relevant regions in glioblastoma have been associated with genotypic characteristics, implying a potential role in personalized medicine. Here, we assess the reproducibility and association with survival of two volumetric segmentation platforms and explore how methodology could impact subsequent interpretation and analysis.

**Methods:**

Post-contrast T1- and T2-weighted FLAIR MR images of 67 TCGA patients were segmented into five distinct compartments (necrosis, contrast-enhancement, FLAIR, post contrast abnormal, and total abnormal tumor volumes) by two quantitative image segmentation platforms - 3D Slicer and a method based on Velocity AI and FSL. We investigated the internal consistency of each platform by correlation statistics, association with survival, and concordance with consensus neuroradiologist ratings using ordinal logistic regression.

**Results:**

We found high correlations between the two platforms for FLAIR, post contrast abnormal, and total abnormal tumor volumes (spearman’s r(67) = 0.952, 0.959, and 0.969 respectively). Only modest agreement was observed for necrosis and contrast-enhancement volumes (r(67) = 0.693 and 0.773 respectively), likely arising from differences in manual and automated segmentation methods of these regions by 3D Slicer and Velocity AI/FSL, respectively. Survival analysis based on AUC revealed significant predictive power of both platforms for the following volumes: contrast-enhancement, post contrast abnormal, and total abnormal tumor volumes. Finally, ordinal logistic regression demonstrated correspondence to manual ratings for several features.

**Conclusion:**

Tumor volume measurements from both volumetric platforms produced highly concordant and reproducible estimates across platforms for general features. As automated or semi-automated volumetric measurements replace manual linear or area measurements, it will become increasingly important to keep in mind that measurement differences between segmentation platforms for more detailed features could influence downstream survival or radio genomic analyses.

## Introduction

Glioblastoma is a highly malignant brain tumor with one of the worst survival rates of all cancers [1]. Standard-of-care treatment typically involves neurosurgical gross total resection, radiation therapy, and temozolomide chemotherapy [2]. However, despite therapeutic advances [3–6], relatively modest progress has been made in the last 20 years in terms of overall survival [7, 8].

The molecular heterogeneity among patients with GBM discovered in large genomic studies suggests that personalized treatment approaches targeting specific pathways may be beneficial [9–11]. With this increasing focus on genomic analysis, new opportunities exist for quantitative radiological imaging to aid in clinical management. However, GBMs differ not only in genomic makeup and microscopic properties, but also in their macroscopic phenotypic properties as observed on MR imaging, which likely reflect both molecular alterations and clinical course [12]. The development of non-invasive biomarkers based on neuroimaging and mining the information embedded in these images that are already routinely collected as part of standard-of-care may better capture the observed heterogeneity and offer valuable insight into cancer biology.

Radiological-genomic correlation studies provide insight into the relationship between tumor genotypes and imaging phenotypes. For example, glioblastoma imaging features have been related to a wide variety of genomic phenomena. *TP53* mutations have been correlated with multifocal gliomas [13] and *IDH1* mutations with non-enhancing tumor [14]. At the transcriptional level, VEGF expression and its prognostic value are affected by the level of edema of the tumor [15], while other imaging features such as tumor contrast enhancement and mass effect have been correlated with hypoxic and proliferation transcriptional patterns [16]. Finally, imaging features such as mixed-nodular enhancement have been correlated with tumor DNA methylation status [17].

The methodologies employed in these studies vary from simple 2D measurements of maximal tumor width on a single axial plane, qualitative assessments for the presence/absence of features of interest (e.g. necrosis, contrast enhancement, DTI signal inhomogeneity), or volumetric/pixel-based approaches. In a previous study, we assessed a set of standardized MR imaging features quantified by three board-certified neuroradiologist reviewing cases according to the VASARI (Visually Accessible Rembrandt Images) standard [18]. The VASARI feature-set consists of 30 features with specific guidelines on how to score each (i.e. 0–5%, 6–33%, 34–67%, 68–95% or > 95% contrast-enhancing), and is designed to allow for accurate and reproducible MR image scoring using a qualitative approach.

One of the challenges is that robust volumetric assessment and tumor segmentation are not a routine part of the radiologists’ regular clinical workflow, nor are these capabilities routinely implemented across the various DICOM workstations. However, as technological advances continue, automated volumetric measurements may eventually supplement manual conventions and offer more accuracy [19]. One area that will likely benefit from more accurate 3D segmentations is *imaging genomics* or *radiomics*, a growing field focused on extracting useful information from radiology imaging data in order to comprehensively quantify the tumor phenotype using advanced imaging algorithms [20–23].

Many tools have been developed to define tumor boundaries and several fully-automated segmentation algorithms have been proposed, such as automated probabilistic segmentation [24, 25], unsupervised fuzzy c-means and nonfuzzy clustering [26], and morphological edge detection and region growing segmentation [27]. Brain Tumor Image Analysis (BraTumIA) is a popular tool for automated tumor segmentation that has been validated in several independent studies, suggesting its potential for patient segmentation or disease monitoring [28, 29]. Semi-automated tumor segmentation, where a computerized segmentation is carried out after an initial manual annotation is made, is often more accurate since it immediately eliminates surrounding tissue that has similar intensity to the tumor but is clearly not malignant [30]. Two important algorithms using a semi-automated segmentation approach for glioblastoma are interactive multi-scale watershed methods that divide brain from tumor based on information from a manual tracing of an initial slice [31] and balloon inflation method [32].

Within the academic and research community, a popular platform for such work is 3D Slicer, a free open-source application for medical image analysis and visualization that, due to its special functionalities, extensibility, and portability across platforms, is actively supported by the National Institutes of Health (NIH) and frequently used by its Quantitative Imaging Network (QIN) [33]. This platform has a number of segmentations algorithms, ranging in complexity and automation. One basic segmentation mechanism uses a “Grow Cut” algorithm based on assigning “seed” pixels that should be segmented together [33–36].

To determine the potential influence of image analysis methods on segmentation, we compared volumetric results obtained from two different platforms: 3D Slicer as well as an in-house method based on Velocity AI (now part of Varian) [37] and the FMRIB Software Library, an image analysis library (FSL) [38]. Using an initial cohort of 67 patients from the TCGA glioblastoma database, we compared these methods to each other through correlation statistics, survival analyses, and agreement to manual ratings obtained through a consensus group of neuroradiologists.

## Materials and Methods

### Patient selection

MR image sets from 67 patients from various institutions downloaded from The Cancer Imaging Archive (TCIA) were used in our analyses. These patients represent a subset of patients who had been recruited from The Cancer Genome Atlas (TCGA) project [39]. Image sets containing both post-gadolinium (Gd) contrast-enhanced T1-weighted (T1w) and T2-FLAIR images were downloaded in DICOM-format and were individually reviewed before feature segmentation to confirm their pre-surgical and treatment-naive status, as well as to exclude images of exceptionally poor quality. As the patients had been previously de-identified by the TCGA and are available for public download, no Institutional Review Board approval was required.

### Volumetric segmentation and measurement

#### Platform 1: 3D Slicer

We used 3D Slicer, a package developed at BWH/Harvard Medical School [40], to initially perform volumetric analysis. The volumes for the 67 patients used in the present analysis were calculated and reported from a subset of 78 volumes used in a previous report [41]. This previous report studied the correlation between edema/cellular invasion molecular patterns and MRI-phenotypes in GBM, whereas the present study investigates the reliability of the segmentation method itself. Segmentation was performed on the images by two board-certified neuroradiologists with over 25 years combined experience in image segmentation (R. Colen, F. Jolesz) to segment whole tumor, necrotic, and contrast-enhancement regions. 3D Slicer has more than 125 modules for image visualization, segmentation, registration, and 3D visualization [42]. T2-FLAIR images were used for calculating peritumoral FLAIR region and post-contrast T1w images for segmentation of the contrast-enhancement and necrosis volume. Spoiled Gradient Echo Recalled (SPGR) images were used for segmentation of the necrosis and contrast-enhancement volume if post-contrast T1w images were not present in the dataset. In patients where FLAIR images were not available, T2w and/or proton density images were used to segment the peritumoral FLAIR volume.

In short, the FLAIR and post-contrast T1w image sets were brought into spatial alignment. Afterwards, a fusion step took place to blend the data in both images into each other so that the three tumor image compartments, namely, peri-lesional edema/infiltration, tumor-enhancement, and necrosis could be delineated and quantified on the same image/slice level. Post-contrast T1w images were used as fixed images and T2-FLAIR as moving images for registration by mutual information optimization (Figure S1). The General Registration (BRAINS) and Transforms module were used for registration. BRAINS is an automatic registration module that provides linear and elastic transforms. The Transforms module requires more user interaction and was used to rigidly and manually align voxels of different volumes in space coordinates until optimal mapping was reached. After images were aligned, the 3D Slicer Editor module was used for segmentation. Abnormal peritumoral FLAIR volume (reflecting edema/invasion), tumor-enhancement, and necrosis volumes were manually color-coded and delineated beginning from the peripheral edema/invasion and going centrally to enhancing and necrotic regions in a single label map approach (Figure S2). The Draw effect module was used to manually and precisely delineate different structures to create colored label maps that were then used to build up 3D models to better visualize spatial relationships between tumor components. The volumes of the delineated tumor compartments were automatically calculated and generated by 3D Slicer Label Statistics module.

#### Platform 2: Velocity AI/FSL

**Figure 1:**
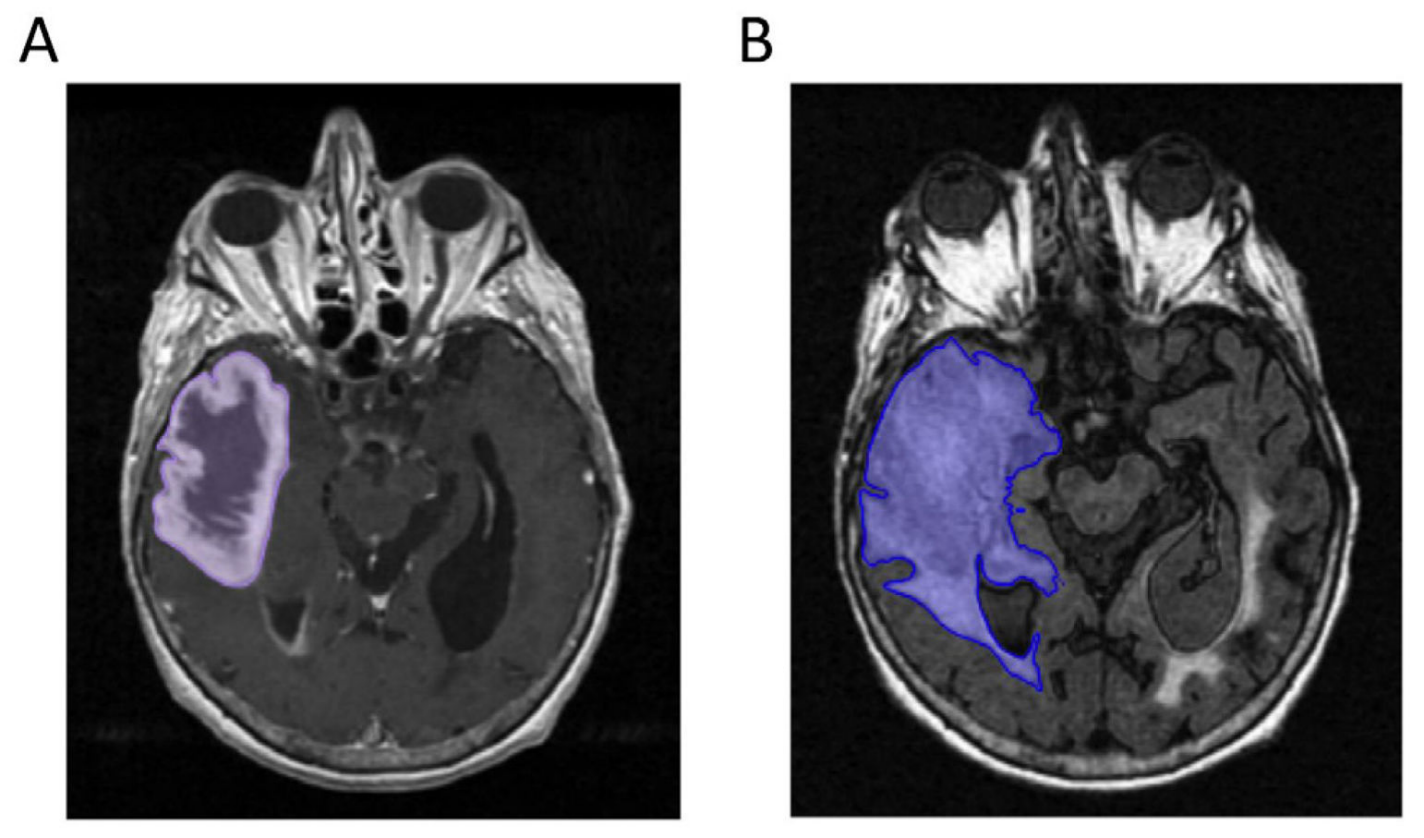
Binary mask segmentation for a 60 year old male patient with a right temporal GBM. Masks were manually drawn in Velocity AI using the 2D flood fill tool (purple, blue areas) to cover tumor volumes on (A) post-contrast T1w images and (B) T2-FLAIR images. Note that the area occupied by the ventricles has been omitted from the T2-FLAIR mask.

The same 67 patients above were also analyzed using a semi-automated approach combining Velocity AI (Atlanta, GA) and FSL (Oxford, UK). Velocity AI, marketed primarily for radiation therapy treatment and image fusion, is specifically designed for the markup/masking and visualization of MR images. For post-contrast T1w images, masks were manually drawn over the tumor and the region it surrounds using a segmentation tool in Velocity AI (Figure 1A). Of note, only a single mask corresponding to the abnormal signal detected on a post-contrast T1w image was drawn; necrosis and contrast-enhancement regions were not delineated at this point.

Similarly, for T2-FLAIR sequences, masks representing the tumor’s total abnormal signal detected by FLAIR were drawn in an analogous manner to the post-contrast T1w images (Figure 1B). This area included all of the areas indicative of edema, as well as other regions that showed signal abnormalities [43]. For the purposes of this study, we did not attempt to differentiate between bright FLAIR signals generated from edema versus non-contrast enhancing tumor. Because we were interested in the FLAIR signal as an estimation of overall tumor involvement, we limited the FLAIR segmentations to regions clearly contiguous with the primary tumor, and did not include for example, thin layers of extension away from the dominant mass along the epididymal surface.

**Figure 2:**
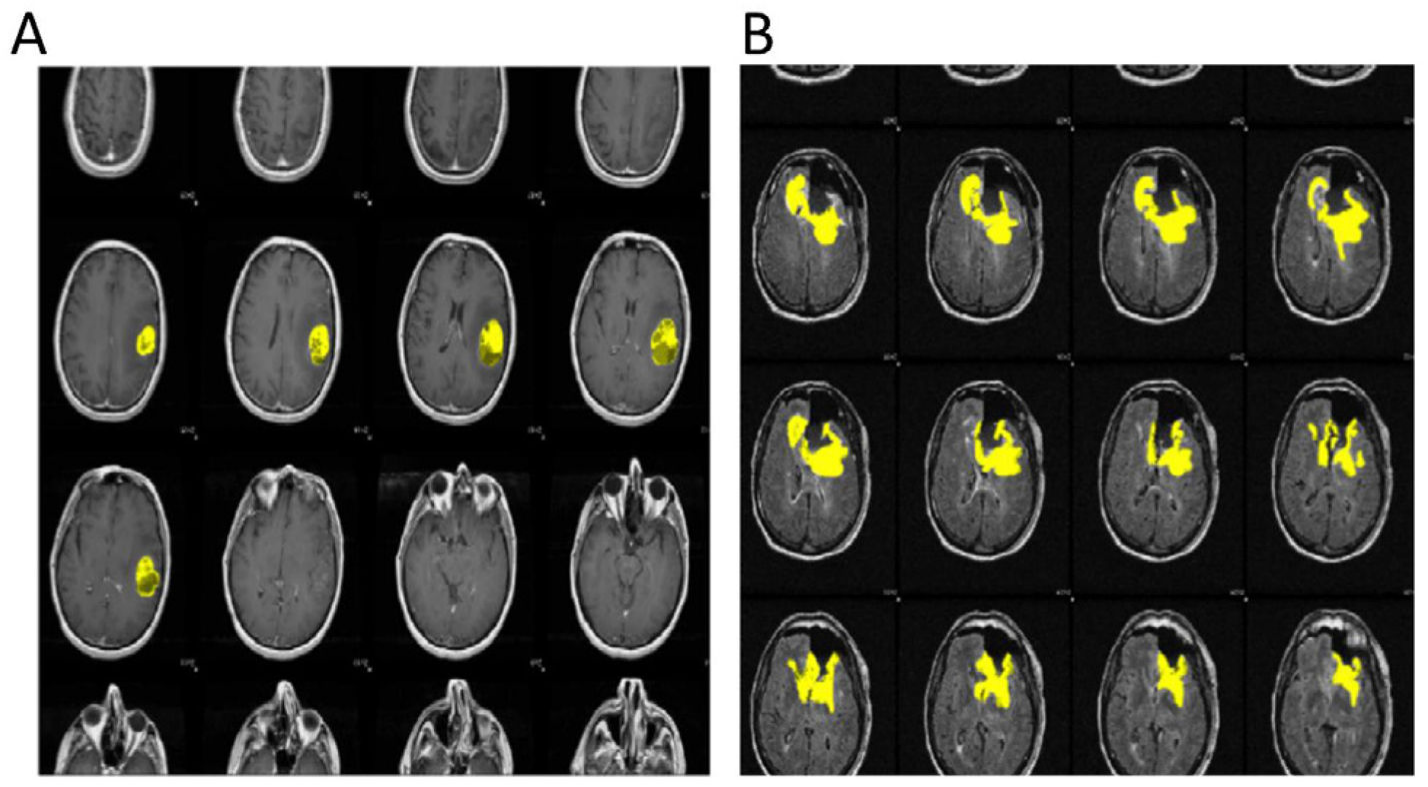
Tumor View Dynamic Reader. Image sets of TCGA patients with accompanying binary masks were uploaded onto an ad hoc website and analyzed. Patients with acceptable images and mask overlay (A) were cleared for analysis whereas patients with poor images or post-surgical images (B) were excluded.

Following initial tumor markup, the masks were exported from Velocity AI as DICOM-RT objects. These image markups were subsequently converted to NIFTI and PNG formats to allow further visualization and segmentation. As a secondary quality check, a custom visualization platform was developed as part of a larger project in our lab (TumorView, Figure 2) that enabled rapid screening of the image volumes that were analyzed for this study. This tool helped eliminate images of particularly poor quality or those from post-surgical image sets (and hence not amenable to estimation of presurgical tumor volume), as well as to ensure that the masks and accompanying images were overlaid and exported properly.

**Figure 3:**
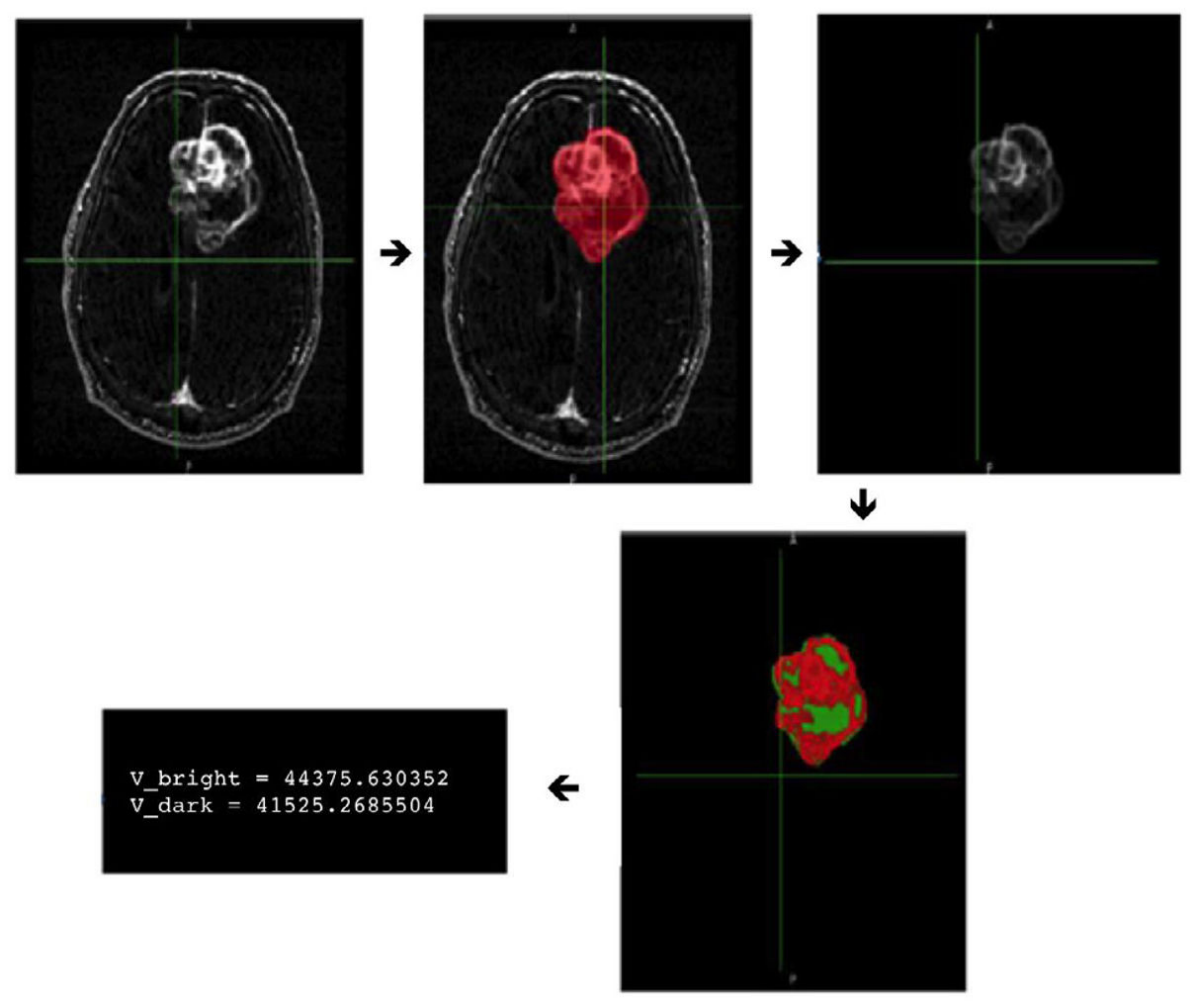
Semi-automated segmentation and measurement method for T1w post-contrast images. Tumor volume was identified by a manually-drawn binary mask (red, second frame) in Velocity AI. K-means clustering divides pixels covered by the mask into bright (red, contrast enhancement) and dark (green, necrosis) clusters based on relative pixel intensity. Volume of individual features can be estimated by converting voxels into mm^3^.

Contrast-enhancement and necrosis volumes were calculated from the masked region on post-contrast T1w images using FAST (FMRIB Automated Segmentation Tool [44], a tool included in FSL). Briefly, for the masks drawn on the post-contrast T1w images, encompassing both contrast-enhancement and necrotic regions, a k-means clustering algorithm was used to identify two clusters based on relative pixel intensity. This resulted in a binary classification of tumor into either bright (e.g. contrast-enhancing) or dark (necrotic) regions (Figure 3). This voxel-based measure could then be directly converted to a volumetric measure by multiplying the number of dark voxels (class 1) or bright voxels (class 2) by the voxel size in mm^3.^

**Figure 4:**
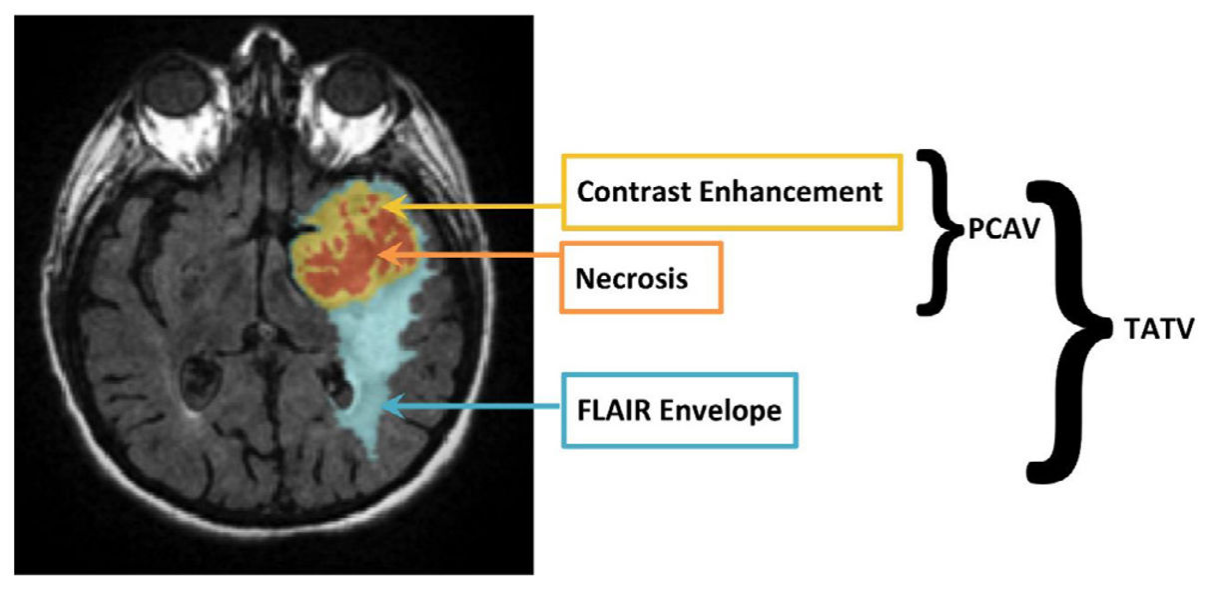
Naming conventions used throughout this article for various tumor compartments. TATV (Total abnormal tumor volume) corresponds to total abnormal signal from the T2-FLAIR scan. PCAV (post contrast abnormal volume) represents the sum of necrosis and contrast-enhancement volumes. FLAIR Envelope represents the difference of PCAV from TATV volume. Base MR image reprinted from [41] under open access license.

For subsequent analysis used throughout this manuscript, we define the post contrast abnormal volume (PCAV) as the sum of the necrosis (NE) and contrast-enhancement (CE) volumes. For the FLAIR images, the entire abnormal signal is referred to as the total abnormal tumor volume (TATV). Finally, the FLAIR envelope refers to the difference between the PCAV and the TATV (Figure 4).

We will refer to 3D Slicer as Platform 1 and Velocity AI/FSL as Platform 2 for the purposes of this article.

#### Consistency of measurements between volumetric images methods

In order to measure the correlation between measurements for various tumor compartments across both platforms, Spearman correlation coefficients were calculated for each of the five volumes. Spearman method was used due to its robustness in approaching outliers as well as to the fact that according to Shapiro-Wilk normality tests, nearly all volume measurements from both platforms did not follow normal distributions. In addition, to view global trends between Platforms 1 and 2, Bland-Altman plots were generated [45]. Bland-Altman plots analyze agreement by comparing the means and differences of each value measured by two different instruments. They are used to compare clinical measurement techniques and are designed to provide more information than simple correlation coefficients.

### Survival analysis

In order to explore the accuracy of our segmenting techniques, we performed several analyses to investigate whether the volumes obtained through both platforms could predict survival. We hypothesized that if our survival correlations were significant using imaging features previously implicated as survival imaging markers in glioblastoma, it would be more probable that these imaging features were accurately segmented. We first assessed whether the mean volumes for various tumor compartments were significantly different from each other when patients are grouped into short-term and long-term survivors. Given the lack of a true gold standard for the volume of any particular tumor subregion, we assessed the ability of each methodology to predict a clinically meaningful endpoint, survival at one year. Patients in the initial 67 patient cohort with survival data were split into groups surviving more than one year (N = 37) and those surviving less than one year (N = 23) and the means of these groups were compared using a Wilcoxon rank-sum test for each imaging feature and several derivative ratios.

After measuring the association between imaging features and survival, we next investigated whether various imaging features were predictive of survival. To quantify prognostic performance of the features for survival prediction, the area under the curve (AUC) of the receiver-operating characteristic (ROC) was assessed using the same 60 patients as the analysis above. For the survival analysis, we predicted one-year survival after date of initial diagnosis.

#### Concordance of volumetric segmentations from both platforms with consensus ratings from neuroradiologists

Finally, we also explored the accuracy of our segmentation techniques by measuring the agreement between the volumes obtained through each platform and those estimated by a consensus group of neuroradiologists rated through the VASARI project. VASARI is a vetted, tested, and validated controlled terminology that aims to comprehensively and reproducibly describe MR imaging [46]. Of the original 67 patient cohort, 59 were also analyzed by neuroradiologists using the VASARI feature-set. Tumors from these 59 patients were analyzed by at least three different neuroradiologists and final scores were based on a consensus between the raters.

In order to compare these resulting categorical scores to our volumetric results, we first identified equivalent variables between the VASARI feature set and our volumetric features: proportion necrosis, proportion contrast-enhancement, and proportion edema. We converted our volumetric features to these VASARI features by dividing necrosis volume, contrast enhancement volume, and FLAIR volume, respectively, by TATV. Next, for each volumetric platform, we measured the association between these quantitative measurements with the categorical measurements based on VASARI (0–5%, 6–33%, 34–67%, 68–95% or >95%) using ordinal logistic regression. Each categorical range was converted to 1, 2, 3, 4, and 5 respectively.

The proportional-odds assumption of our models was verified by visually inspecting that the empirical odds ratios between larger versus smaller VASARI scores and predictor variables was relatively constant over the levels, in a number of cases checked, and thus considered valid to the extent possible given our data. For significance, we used the Wald Chi-Square test to test the null hypotheses that all the fitted coefficients in the model are zero, i.e. the quantitative volumetric measures do not predict the trend.

## Results

### Consistency of measurements between volumetric images methods

First, we investigated the correlation between both volumetric techniques for estimation of total abnormal tumor volume (TATV), post contrast abnormal volume (PCAV), FLAIR envelope volume, necrosis volume, and contrast-enhancement volume.

We found strong Spearman’s rank correlation coefficients between both platforms for measuring TATV and PCAV (r(67) = 0.97 and 0.96 respectively) (Table 1). Strong correlations were also observed for FLAIR envelope volume (r(67) = 0.95). Fair to good correlations of 0.693 and 0.773 were observed for both platforms for estimating necrosis volume and contrast-enhancement volume respectively.

**Figure 5:**
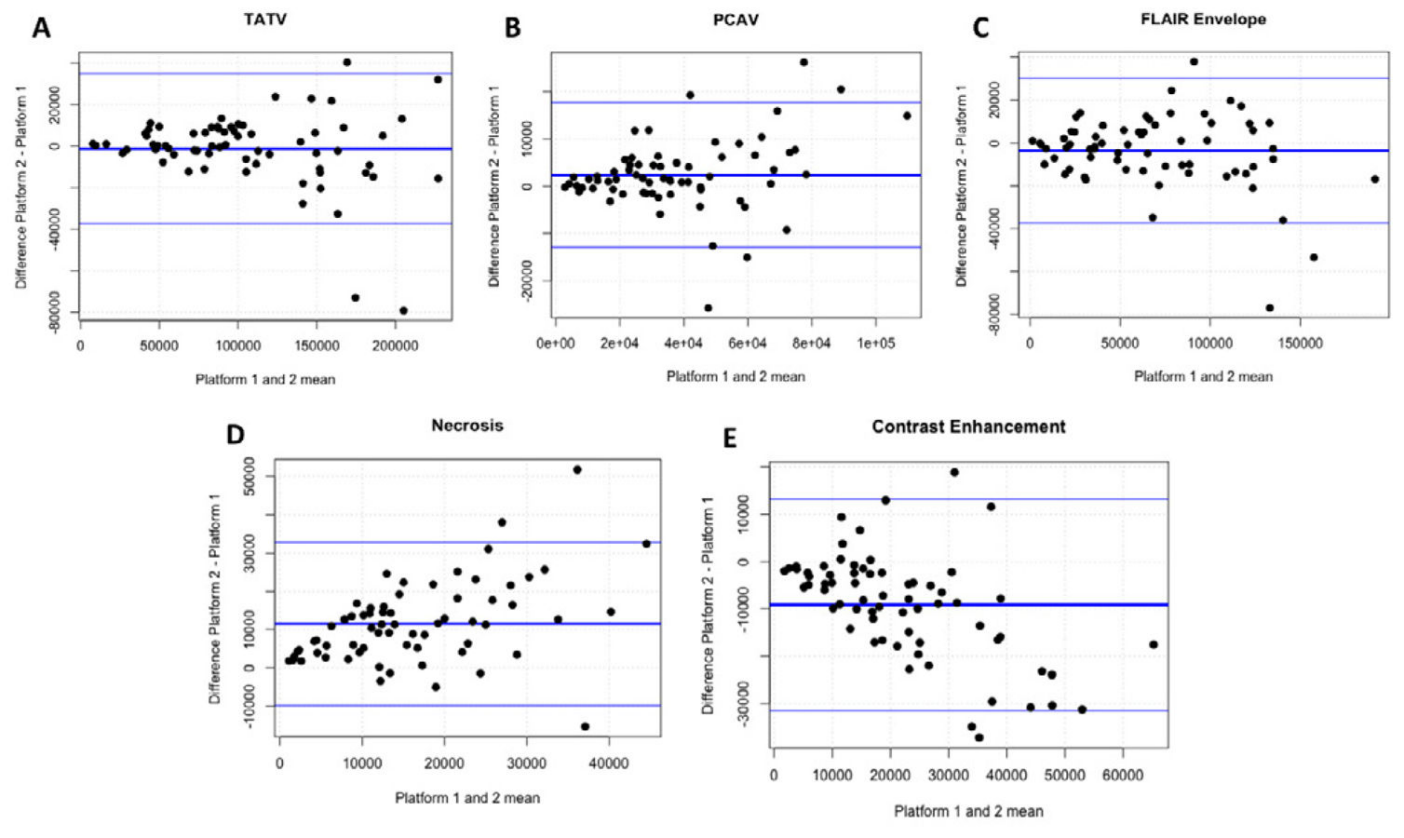
Bland-Altman plots showing the measurement trends for each imaging feature. In each graph, the middle horizontal bar represents the average difference of the measurements obtained from Platform 1 subtracted from those of Platform 2 for all patients measured (N = 67). A mean of 0 suggests no bias between the two platforms. The upper and lower horizontal bars represent 1 standard deviation from the average difference. According to literature consensus, points that fall outside of these lines significantly differ between measurements. Total abnormal volume (A), post contrast abnormal volume (B), FLAIR Envelope (C), necrosis volume (D), and contrast-enhancing volume (E).

Bland-Altman plots were also generated to evaluate these patterns more in depth (Figure 5). In general, the differences in measurements obtained by the two platforms increased as their resulting volumes increased. Near zero mean differences between both platforms for TATV, PACV and FLAIR envelope volume suggests that no systematic platform bias exists. However, the delineations between contrast-enhancing volume and tumor necrotic volume were relatively less congruent. For example, Platform 1 consistently identified more contrast enhancement and less necrosis volume than did Platform 2.

### Survival analysis

As no gold standard exists for tumor volume, we chose a secondary endpoint to assess the relative robustness of these two volumetric methods. For simplicity, we first generated a cohort of patients with survival greater than one year (N = 37) or less than one year (N = 23). Using volumes obtained from Platform 2 (Velocity AI/FSL), patients surviving less than a year were found to have on average significantly greater volumes of necrosis (*P* = 0.0027), contrast-enhancement (*P* = 0.0094), post-contrast abnormal volume (*P* = 0.0041), and total abnormal tumor volume (*P* = 0.0267) than those surviving more than a year (Table 2). Measurements obtained by Platform 1 showed similar trends and significances, except with regards to necrosis volume (*P* = 0.0975).

The prognostic performance of the volumes was assessed using the area under the curve (AUC) of the receiver operating characteristic (ROC). For both platforms, contrast-enhancement, PCAV, and TATV, were measured to be significantly prognostic (Table 3). Furthermore, similar to above, Platform 2 additionally quantified necrosis as a strong prognostic parameter, with an AUC > 0.7 (*P* = 0.0008). For both platforms, no significance was found for the FLAIR envelope volume, Necrosis/TATV, Contrast-Enhancement/TATV, and FLAIR Envelope/TATV, indicating the limited prognostic value of these features.

#### Concordance of volumetric segmentations from both platforms with consensus ratings from neuroradiologist

Finally, ordinal logistic regression results are displayed in Table 4. P values for Wald Chi-Square tests show that Platform 1 showed consistent agreement with all three scores derived using the VASARI criteria, while Platform 2 showed agreement with only proportion of edema.

## Discussion

In this work, we compared two volumetric segmentation approaches based on Velocity AI/FSL and 3D Slicer and demonstrated that both platforms produced very consistent estimates of TATV and PCAV (r(67) = 0.97 and 0.96 respectively). While estimates of sub-compartment volumes (necrosis and contrast enhancement) were also significantly correlated, these correlations were much less robust (r(67) = 0.69 and 0.77 respectively). In the case of 3D Slicer, these compartments were estimated by manual contouring, and the sum of those two sub compartments would then equate to the PCAV. For estimates of NE and CE based on the Velocity AI/FSL approach, we instead relied on automated k-means clustering to semi-automatically segment the PCAV into the two compartments (bright/dark → CE/NE).

### Technical explanation for segmentation discrepancies

Given the relationship between NE and CE, we generated Bland-Altman plots to look for any systematic differences between these results to assess for a bias. Bland-Altman plots indicated Platform 1 consistently labeled more voxels as contrast-enhancement (and fewer as necrosis) than Platform 2. These results suggest that not only is there a loss of reproducibility between platforms for smaller sub-regions compared to larger regions, but also that there is a systematic difference rather than a difference attributable to random chance. It is important to note these systematic differences as they could translate to systematic differences in downstream survival or biological analyses.

The differences in segmentation results for these smaller sub-regions were likely a result of the inherent k-means clustering algorithm used in Platform 2 that clusters pixels based on intensities using a cutoff derived from that specific intensity volume histogram. The threshold between what determines whether a voxel is labeled as contrast-enhancement or necrosis is also fairly subjective in the case of manual delineation in Platform 1, whereas a k-means method is based purely on the raw values fed into the cluster algorithm, and therefore not subject to the brightness/contrast and windowing settings of the workstation that could impact human segmentation.

It is important to take into consideration that the data set we used was collected from a consortium of institutions within the TCGA network using different MR scanners, protocols, and pulse sequences which all add significant variability in signal-to-noise ratios (SNR) and what appears to be contrast-enhancement/necrosis regions [9]. This would prevent any reasonable attempts to try and normalize the window level used for threshold in Platform 2 that could potentially obviate that effect.

### Survival analysis

Using one-year survival as an endpoint, both platforms demonstrated that patients with more contrast-enhancement, PCAV, and TATV showed significantly shorter survival times. A more sophisticated model using AUC analyses also demonstrated that these same measures were associated with survival. Interestingly, for both types of analyses investigated, Platform 2 additionally measured necrosis volume as being a significant predictor of survival.

The results from our survival analysis suggest that both platforms accurately identify gross overall features such as TATV and PCAV as previous research has demonstrated association between metrics that measure overall tumor and survival [47, 48]. In addition, volume of contrast-enhancement, representing neovascularity and angiogenesis, has also been implicated as being significantly associated with overall survival through qualitative [49, 50] and quantitative [48] studies.

While necrosis has also typically been implicated in survival [50, 51], other studies have not found significant associations. For example, in a study, based in part on the patients used in the present analysis, necrosis was not found to be associated with survival. Colen et al. [52] suggested that this discrepancy may be related to patient sex: necrosis was only significantly associated with survival for females, likely due to differences in MYC and TP53 in cell death between males and females [52].

We should note previous studies have suggested that feature to survival correlation likely does not follow a linear model [50]. In addition, although the quantified features showed strong performance (AUC~0.70), these results have to be validated in larger cohorts to determine true prognostic performance. However, the main goal of these analyses, rather than to thoroughly assess the relationship between specific imaging features and survival, was to investigate whether both platforms produced similar results.

### Segmentation accuracy

Our work evaluates the reproducibility of the two segmentation platforms in regards to their ability to consistently segment the same regions. Our results suggest that the segmentation technique can lead to different measurements in certain cases that may ultimately influence survival prediction and radio-genomic correlations.

A related but different concept to reproducibility is accuracy, or how close the measured value is to the “true” number within the specific region of interest (ROI). Indeed, the task of choosing a gold standard to objectively measure accuracy was a challenge throughout this project and a repeating issue in the field in general. To explore this question, we used various markers at our disposal to measure accuracy. For instance, a number of imaging features have been shown to be significantly associated with overall survival, which is both an unambiguous endpoint and completely independent of the imaging metrics. Thus, we could consider our measurements “accurate” if we could reproduce these associations with the measurements using our two platforms. However, this approach is only valid if literature already consistently demonstrates the features have been associated with survival; in the case of many potential radiological features this is not the case. We also explored accuracy through measuring agreement with radiologists’ measurements of related features using the qualitative VASARI method. This is also not a perfect marker of accuracy since the VASARI results were influenced by inter-rater variability and while experts performed the ratings, visual estimations of tumor volume from a set of 2D images is an inherently difficult task.

The implications of accurate segmentations vary depending on the specific use case and in many cases necessitate that the investigators weigh the relative advantages/disadvantages of the various approaches (e.g. manual segmentation takes longer, but may result in segmentations with improved accuracy). The ability to obtain accurate volumetric measurements is important in a variety of clinical or research applications. For example, in a direct clinical setting, accurate volumetric measurements are important for surgical and radiotherapy guidance and planning, and to a lesser extent, for surveillance during clinical trials. In regards to research with downstream clinical implications, accurate segmentations are important when the volumes and masks serve as the basis for subsequent radiogenomic analysis. It is likely that more accurate segmentations will more precisely target feature of interest that have common molecular or genetic bases from confounding features and lead to more reproducible associations. Imaging genomics is a growing field especially in the cancer domain [41, 53, 54] and has the potential to expand into different areas such as neurology and public health [55]. Our results suggest that, in order for volumetric MRI analysis programs such as 3D Slicer to continue to play a strong role in the future of imaging genomics, as well as more traditional roles of prognosis, staging, and response assessment, special focus is required to ensure accurate segmentation measurements. Therefore, future work will more strongly establish survival-associated imaging markers through consistent results based on large-sample studies as well as incorporate other potential markers for accuracy such as treatment response or relapse of the tumor during surgery.

## Conclusion

As quantitative volumetric image analysis gains an ever-increasing foothold in clinical and research domains, we are likely to benefit by gaining a stronger insight into the biology of glioblastoma, as well as other oncological or neurological diseases. Many volumetric imaging platforms already exist - open source, licensed, automated, semi-automated, etc. – and are based on a wide range of underlying techniques. Our results suggest that certain features are robust across platforms, particularly those related to the total area of abnormal signal. Likewise, since measurements for more detailed sub volumes varied more between platforms, results from downstream radiogenomic analyses should be interpreted more carefully until these volumetric techniques are thoroughly validated.

## Supplementary Material

Supplement Figures

## Figures and Tables

**Table 1:** Correlation results from volumes measured between platform 1 and 2 (N = 67).

Feature	Spearman rho between platform 1 and 2	p-value rho ≠ 0
Necrosis (mm^3^)	0.693	8.398E-11*
Contrast Enhancement (mm^3^)	0.773	1.807E-14*
FLAIR Envelope (mm^3^)	0.952	2.2E-16*
PCAV (mm^3^)	0.959	2.2E-16*
TATV (mm^3^)	0.969	2.2E-16*

Significant P values (< 0.05) indicated by *

**Table 2:** Comparison of means of volumes and ratios between patients surviving less than one year (N = 23) and those surviving more than one year (N = 37) for various imaging features. Same patients were used across both volumetric platforms. Significance values reflect Wilcoxon rank-sum test.

	Platform 1: 3D Slicer			Platform 2: Velocity A1		
Imaging Feature	x̄ < 1 yr	x̄ > 1 yr	Sig.	x̄ < 1 yr	x̄ > 1 yr	Sig.
Necrosis (mm^3^)	1.33E+04	8.50E+03	9.75E-02	2.78E+04	1.76E+04	2.70E-03*
Contrast Enhancement (mm^3^)	3.34E+04	2.04E+04	2.06E-03*	2.18E+04	1.39E+04	9.42E-03*
FLAIR Envelope (mm^3^)	7.74E+04	6.10E+04	3.57E-01	6.91E+04	5.95E+04	3.49E-01
PCAV (mm^3^)	4.67E+04	2.89E+04	2.06E-03*	4.96E+04	3.15E+04	4.11E-03*
TATV (mm^3^)	1.24E+05	8.99E+04	2.36E-02*	1.19E+05	9.10E+04	2.67E-02*
Necrosis/TATV	1.31E-01	1.05E+01	8.43E-01	2.46E-01	2.14E+01	5.16E-01
Contrast Enhancement/TATV	2.81E-01	2.47E+01	4.23E-01	1.96E-01	1.68E+01	6.51E-01
FLAIR Envelope/TATV	5.88E-01	6.48E+01	5.66E-01	5.58E-01	6.18E+01	6.08E-01
PCAV/TATV	4.12E-01	3.52E-01	5.66E-01	4.42E-01	3.82E-01	6.08E-01
Necrosis/Contrast Enhancement	4.68E-01	4.88E-01	8.55E-01	1.35E+00	1.39E+00	7.40E-01
Contrast Enhancement/PCAV	7.38E-01	7.27E-01	8.55E-01	4.34E-01	4.29E-01	7.40E-01

Significant P values (P < 0.05) are indicated by *

**Table 3:** Area Under the Curve (AUC) statistic calculated for each volumetric compartment and its derivative ratios measured for both platforms to predict one year survival.

	Platform 1: 3D Slicer		Platform 2: Velocity A1	
Imaging Feature	AUC	Sig.	AUC	Sig.
Necrosis (mm^3^)	0.6287	6.67E-002	0.7286	8.01E-004*
Contrast Enhancement (mm^3^)	0.7344	2.51E-004*	0.6992	5.30E-003*
FLAIR Envelope (mm^3^)	0.5723	3.49E-001	0.5734	3.25E-001
PCAV (mm^3^)	0.7344	6.05E-004*	0.7192	1.91E-003*
TATV (mm^3^)	0.6745	9.06E-003*	0.6710	1.12E-002*
Necrosis/TATV	0.5159	8.31E-001	0.5511	4.95E-001
Contrast Enhancement/TATV	0.5629	3.80E-001	0.5358	6.39E-001
FLAIR Envelope/TATV	0.5452	5.55E-001	0.5405	5.96E-001

Significant P values (P < 0.05) are indicated by *

**Table 4:** Ordinal logistic regression measuring agreement between each Platform 1 and Platform 2 and gold standard rating using features derived from the VASARI scale. ‘Prop’ refers to the proportion of contrast-enhancement (CE), necrosis (NE), or Edema over TATV. Coef = coefficient of regression, SE = standard error.

		Coef	SE	P
**Platform 1**	Prop CE	1.08E-01	2.97E-02	2.91E-04*
	Prop NE	1.66E-01	4.10E-02	5.20E-05*
	Prop Edema	7.79E-02	1.82E-02	1.87E-05*
**Platform 2**	Prop CE	−8.09E-03	2.74E-02	7.68E-01
	Prop NE	4.61E-02	2.33E-02	4.76E-02
	Prop Edema	5.19E-02	1.41E-02	2.43E-04*

Significant P values corrected by bonferroni (P < 0.015) are indicated by *
