# Supplementary material for: Assessing the Effects of Software Platforms on Volumetric Segmentation of Glioblastoma
Source: J Neuroimaging Psychiatry Neurol. Author manuscript; Available in PMC 2018 Mar 27. (PMC5870135; doi:10.17756/jnpn.2016-008)
Supplement: Supplement Figures [file NIHMS815798-supplement-Supplement_Figures.docx]

**
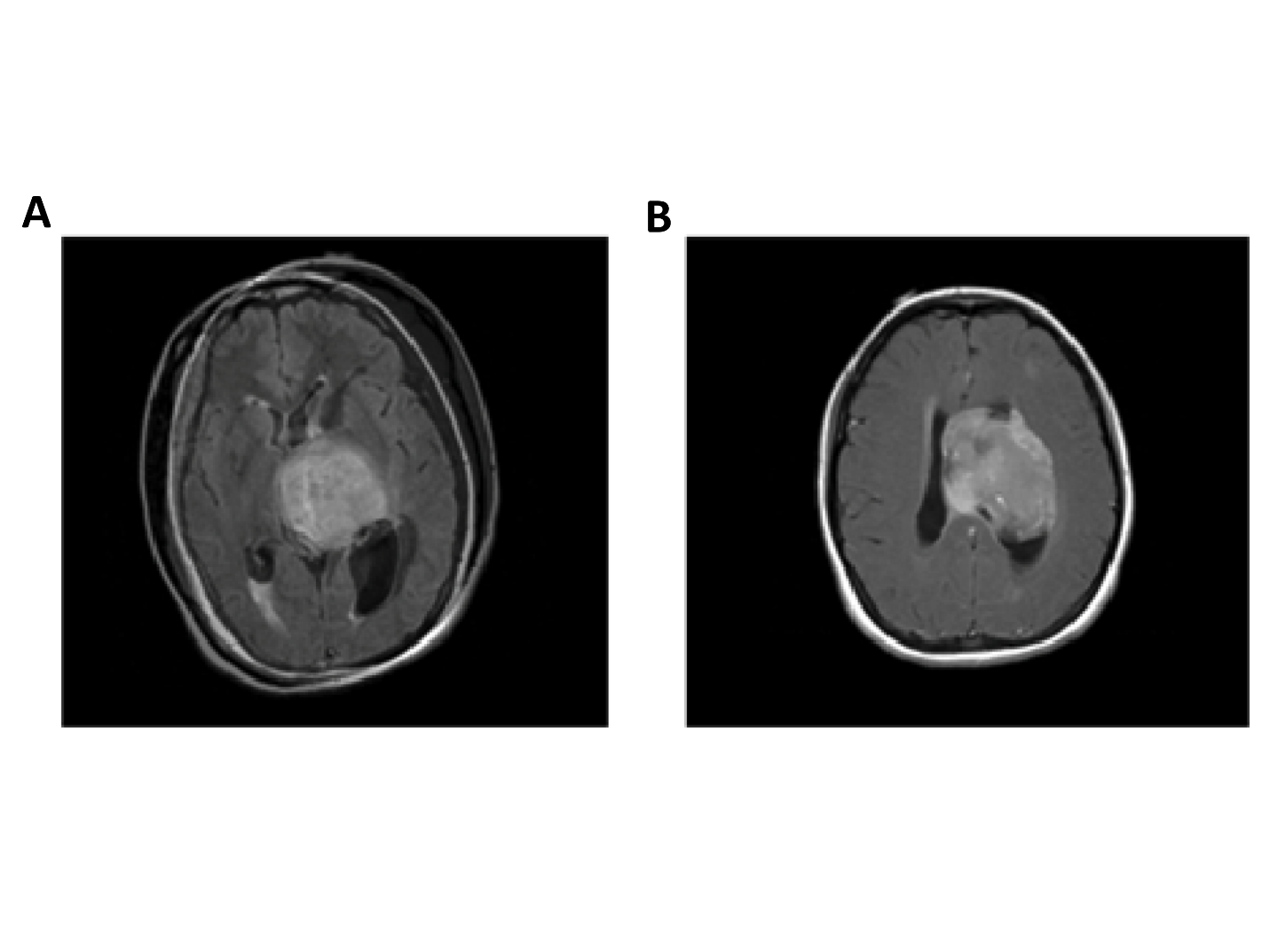
Figure S1:** MR image alignment. (A) Demonstrates post-contrast T1w and T2-FLAIR sequences of similar image spacing that are clearly not aligned. (B) Shows the two images perfectly co-registered using 3D Slicer General Registration (BRAINS) module.

**
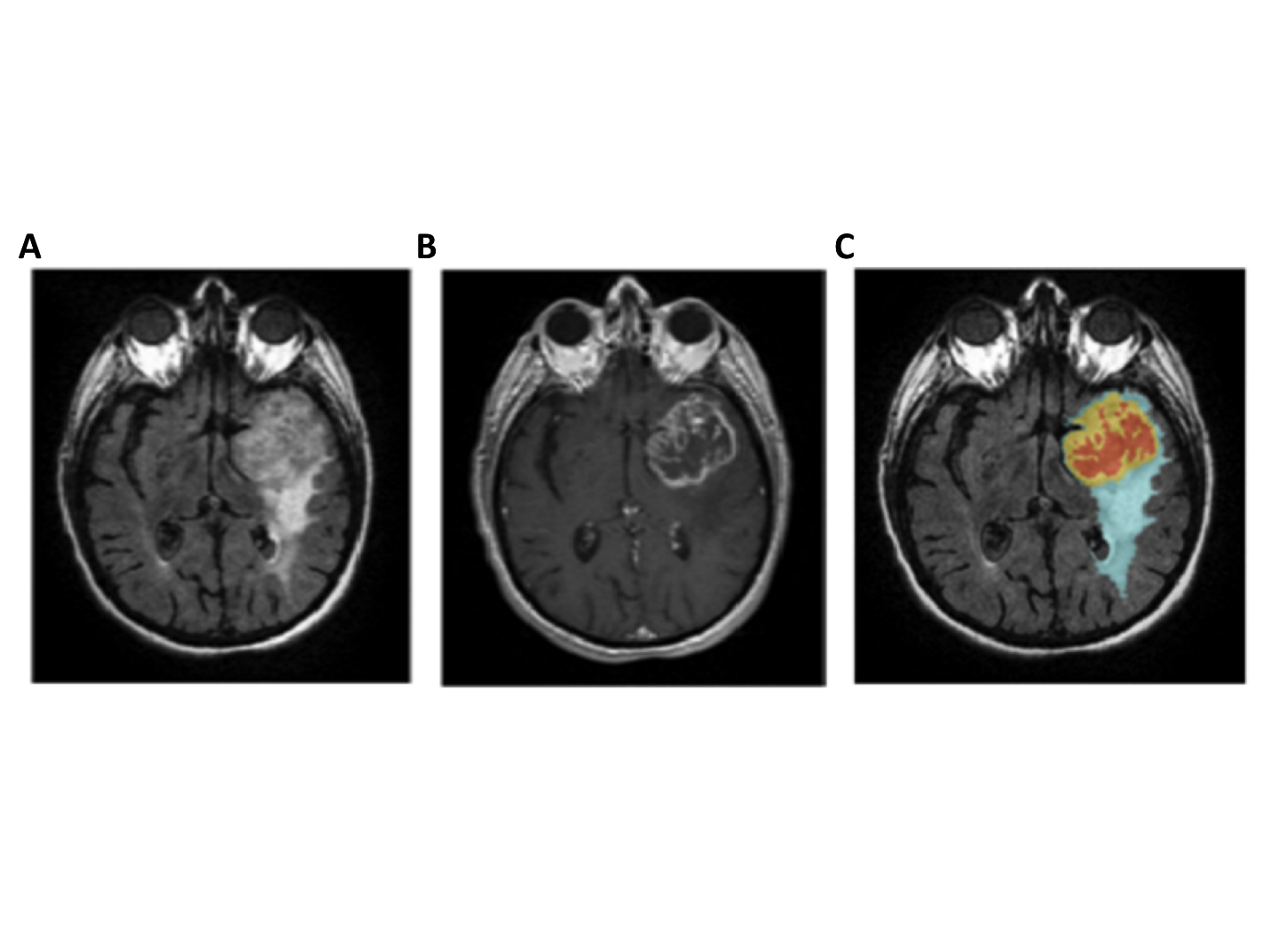
Figure S2:** Axial T2-FLAIR (A) and post-contrast T1w image (B) of patient with GBM. Following co-registration, FLAIR signal, tumor enhancement, and necrosis were manually delineated and color-coded in the single label map approach using 3D Slicer Editor Module (C).
